# Sex differences in excess mortality during the COVID-19 pandemic: a longitudinal ecological analysis of 34 high-income countries

**DOI:** 10.1016/j.eclinm.2026.104072

**Published:** 2026-07-23

**Authors:** Katarzyna Doniec, Jonas Schöley, Mine Kühn, Jennifer Beam Dowd

**Affiliations:** a Leverhulme Centre for Demographic Science, Nuffield Department of Population Health, Nuffield College, University of Oxford, 42-43 Park End Street, Oxford OX1 1JD, United Kingdom; b The Max Planck Institute for Demographic Research, Konrad-Zuse-Straße 1, Rostock 18057, Germany; c Department of Sociology, School of Social and Behavioural Sciences, Tilburg University, Tilburg 5000 LE, the Netherlands; d The Einstein Center Population Diversity (ECPD), Berlin, Germany

**Keywords:** Mortality, Sex differences, COVID-19

## Abstract

**Background:**

The COVID-19 pandemic was a significant global mortality shock. In addition to baseline differences in mortality risk, males often face a survival disadvantage in crisis situations such as famines and epidemics. Although many countries have reported higher numbers of male COVID-19 deaths, the evolution of sex differences in mortality over the course of the pandemic, and how these differences compare using both relative (accounting for higher male baseline mortality) and absolute measures remains underexplored. This study aimed to assess absolute and relative sex differences in all-cause excess mortality during the COVID-19 pandemic across three pandemic phases in 34 high-income countries.

**Methods:**

We examined sex differences in excess mortality from February 2020 to July 2023 using data from 34 countries in the Short-Term Mortality Fluctuations dataset. We estimate both absolute (male excess mortality—female excess mortality) and relative (male P-score—female P-score) sex differences in pandemic-related all-cause excess mortality. We analysed sex differences monthly and across three pandemic phases: pre-vaccine, post-vaccine, and endemic.

**Findings:**

In most countries, absolute male excess mortality exceeded that of females during the pre-vaccine phase of the pandemic. A significant absolute male disadvantage in excess mortality rates was observed in 60% (81/136) of age-group–country combinations in the pre-vaccine phase, decreasing to 51% (70/136) in the post-vaccine phase and 23% (31/136) in the endemic phase. In contrast, a male disadvantage was less common for relative changes: a significant male disadvantage in excess mortality P-scores was observed in only 28% (38/136) of age-group–country combinations in the pre-vaccine phase and 14% (19/136) in the post-vaccine phase. The remaining combinations showed either no significant sex difference or a female disadvantage, with little evidence of relative sex differences during the endemic period. Sex differences in absolute excess mortality tended to grow with age, but there was no clear pattern in the relative sex differences across age groups.

**Interpretation:**

While previous research has highlighted a male disadvantage in COVID-19 mortality based on absolute death counts, our study shows that this pattern was concentrated in the pre-vaccine phase and declined over time. Relative increases in all-cause excess mortality were often similar between sexes and, in some cases, greater among women. These findings suggest that sex differences in pandemic-related mortality were more varied than commonly assumed and underscore the importance of using both absolute and relative measures to assess the impact of health crises on population subgroups. Finally, our results suggest that COVID-19 did not produce lasting shifts in sex differences in mortality.

**Funding:**

This work was supported by the 10.13039/501100000781European Research Council (ERC-2021-CoG-101002587) and the 10.13039/501100000275Leverhulme Trust (Grant RC-2018-003) for the Leverhulme Centre for Demographic Science.

**Research in context**

**Evidence before this study**

We searched PubMed for articles published between March 1, 2020, and March 1, 2026, on sex differences in COVID-19 mortality or excess mortality using the terms (“sex” OR “gender”) AND (“gaps” OR “differences”) AND (“COVID-19” OR “excess mortality”) AND (“mortality” OR “death”). Reference lists of relevant studies were also screened. Existing literature indicates, after standardisation for age, a male disadvantage in COVID-19 mortality, with some variation in high-income countries. Most previous studies relied on official COVID-19 death counts, focused on the early pandemic period, and used absolute measures of sex differences (e.g., differences in death counts or mortality rates). Few studies have applied an excess mortality framework, which allows more direct cross-country and cross-period comparisons. Evidence reporting both absolute and relative measures of sex differences is also scarce. It remains unclear whether male disadvantage persisted across different stages of the pandemic—particularly after vaccine introduction—and whether it was present in all age groups and countries.

**Added value of this study**

We conducted a comprehensive analysis of absolute and relative sex differences in all-cause excess mortality over time, both monthly and across three pandemic stages (pre-vaccine, post-vaccine, and endemic) in 34 high-income countries. We found that male mortality disadvantage was not universal but context-specific and temporary. In most countries, the absolute male mortality disadvantage was concentrated in the pre-vaccine phase, during peak mortality months, and diminished in later stages. The largest absolute male disadvantage was observed in countries with historically greater sex differences in all-cause mortality (Eastern Europe). When using relative measures of sex differences—which account for baseline mortality differences—the male disadvantage was largely absent or reversed in most age group–country combinations. This suggests that earlier conclusions suggesting men were universally more affected by the pandemic in high income countries, were incomplete.

**Implications of all the available evidence**

Building on the studies that have identified a male mortality disadvantage early in the pandemic, our study indicates that this disadvantage was primarily evident when using absolute measures of sex differences, during mortality peaks and diminished over time. A comprehensive assessment of the mortality impacts of health emergencies on different populations requires the use of both absolute and relative measures of mortality differences. Analysis of cross-country sex differences highlighted that the pandemic amplified pre-pandemic sex differences in all-cause mortality, consistent with broader evidence of its magnifying effect on pre-pandemic inequalities.

## Introduction

In modern populations, females generally have lower all-cause mortality rates than males. These sex differences in mortality have been narrowing in recent decades in high-income countries,^1^, ^2^, ^3^, ^4^, ^5^ a trend that may have been disrupted by the COVID-19 pandemic. Globally, higher male COVID-19 mortality was observed early in the pandemic.^6^^,^^7^ Historically, males often have a survival disadvantage during crises such as famines and epidemics,^8^ often attributed to both biological and sociocultural factors.

For COVID-19, there are hypothesised sex differences in immune response and biological susceptibility.^9^, ^10^, ^11^, ^12^, ^13^, ^14^, ^15^, ^16^, ^17^ For example, women tend to have stronger and longer-lasting immune responses than men, partly due to hormonal factors like oestrogen and X-linked genetic traits, which may offer them an advantage in fighting infection. Additionally, social, occupational, and behavioural differences may have influenced exposure to the virus.^18^^,^^19^ Indeed, previous work has shown a male disadvantage in COVID-19 mortality and that the magnitude of sex differences varied by age^20^, ^21^, ^22^, ^23^, ^24^, ^25^ and region.^19^^,^^20^^,^^23^^,^^26^, ^27^, ^28^, ^29^, ^30^, ^31^, ^32^

Previous studies on sex differences in COVID-19 mortality have several limitations. First, most previous studies focused on data from only the early stages of the pandemic. How sex differences evolved over the course of the pandemic and the potential effect on long-term sex differences in mortality^33^ is less known. Biological and behavioural susceptibility to COVID-19 likely evolved over time in ways that could impact sex differences but are hard to predict a priori. On the one hand, vaccines and improved treatments could reduce male biological disadvantage. On the other hand, differential uptake of COVID-19 prevention measures, such as mask-wearing, social distancing, and vaccines, could drive persistent or growing male disadvantage.^34^^,^^35^

Second, previous studies estimated sex differences using absolute numbers of COVID-19 deaths or mortality rates. But since men have higher baseline mortality at each age, any proportionate increase in the risk of death would naturally result in more male deaths. For this reason, it is perhaps not surprising that males experienced a higher number of COVID-19 deaths. However, a higher number of deaths alone doesn’t tell us whether men are more susceptible to the SARS-CoV-2 virus itself, for example. In contrast, relative measures of sex differences -- such as whether one sex is seeing a higher *percentage* increase in deaths compared to expected levels-- can tell us whether sex differences in COVID-19 mortality are worse than for other causes of death. While there is no “correct” metric for quantifying sex differences, a comprehensive assessment of health inequalities can benefit from considering both absolute and relative perspectives.^36^, ^37^, ^38^, ^39^, ^40^

Third, most previous studies of sex differences in COVID-19 mortality rely on official COVID-19 deaths data. However, variations in testing practices and the accuracy of recording COVID-19 as a cause of death make cross-country comparisons challenging.^41^ By contrast, all-cause mortality is more reliably recorded and offers better comparability.^42^, ^43^, ^44^ Furthermore, examining excess mortality—the difference in observed versus expected all-cause mortality based on prior trends— allows us to contextualise sex differences in COVID-19 mortality within pre-pandemic sex differences in each country.

We address these limitations by conducting the first comprehensive examination of trends in sex differences in excess mortality from February 2020 to July 2023, using data from 34 high-income countries. Specifically, we estimate both sex differences in excess mortality death rates and percent excess deaths by month and examine these trends across three key phases of the pandemic: pre-vaccine (February 2020–April 2021), post-vaccine Delta/Omicron (May 2021–April 2022), and endemic (May 2022–July 2023). Additionally, we explore how these sex differences vary by age and country.

## Methods

This manuscript was prepared in accordance with the STrengthening the Reporting of OBservational studies in Epidemiology (STROBE) guidelines. We used the Short-Term Mortality Fluctuations (STMF) dataset for 34 countries to estimate sex-specific all-cause excess monthly mortality between February 2020 through July 2023. This approach has previously been used to estimate the death toll from other mortality crises.^45^, ^46^, ^47^ While excess mortality may also pick up indirect deaths from non-COVID causes during the pandemic, recent evidence from the US suggests that a large fraction of non-COVID-19 excess deaths were likely undercounted COVID-19 deaths.^48^ We estimated the expected (baseline) deaths for each week using the 7 years from 2013 through 2019 as the reference period from which to extrapolate a counterfactual non-pandemic trend. The STMF dataset does not include information on race or ethnicity, as it consists of aggregated national death counts.

Population denominators were sourced from the Human Mortality Database (2025). Person-weeks of exposure were calculated by cubic interpolation of the January 1st population over the weeks of a year, followed by integration of the cubic spline over single weeks. Age-specific person-weeks of exposure were linearly extrapolated by up to two years if no estimates were available.

We used a Generalised Additive Model (GAM) with smooth seasonality and a log-linear time trend to estimate expected deaths. The model was defined as

$$Y_{tw}∼\;\text{Negative}-\text{Binomial}\left(\lambda_{tw},\phi \right)\text{,}$$

with

$$\log\left(\lambda_{tw}\right)=\beta_{0}+\beta_{t}\text{weeks}-\text{since}-\text{origin}+s\left(\text{week}-\text{of}-\text{year}\text{;}\;\theta_{w}\right)+\log\left({\text{person}-\text{weeks}}_{tw}\right)\text{,}$$

where *Y*_*tw*_ -- the number of deaths in week-of-year *w* at time *t*, measured in weeks since 2013 -- is distributed according to a Negative Binomial distribution with expected number of deaths $\lambda_{tw}$ and overdispersion ϕ. Expected deaths were modelled via a log-linear long-term trend and a smooth function of week of year, *s*, implemented as cyclical cubic splines. Logged person-weeks of exposure were added as an offset to model on a death rate scale. The model was fit separately for each country, sex, and age group (0–15, 15–64, 65–74, 75–84, 85+). These age groupings reflect harmonisation choices made by the STMF team to ensure comparability across countries and periods, given substantial heterogeneity in the age classifications of weekly mortality data reported by national statistical offices. Disaggregating the 15–64 group further is not possible in a consistent manner for all countries without relying on model-based ungrouping of weekly death counts, which may introduce bias, particularly during periods of rapid, age-specific mortality change.^49^

Expected deaths were derived by evaluating $\lambda_{tw}$ over the period from February 2020 through July 2023. We aggregated weekly expected and observed deaths to months and across three pandemic periods: pre-vaccine (February 2020–April 2021), post-vaccine Delta/Omicron (May 2021–April 2022), and endemic (May 2022–July 2023).

To quantify uncertainty, we used simulation-based prediction intervals. First, we employed a Normal-approximation to the parametric bootstrap to capture parameter uncertainty.^50^ We sampled parameter vectors from a multivariate Normal distribution derived from the Hessian matrix of the maximum likelihood fit. Given the sampled parameters we generated 250 repeated draws from the predictive distribution of deaths. For each simulated dataset, we recalculated expected deaths, excess deaths, P-scores, and sex differences following the same aggregation steps applied to the observed data. Prediction intervals were defined as the 2.5th and 97.5th percentiles of these simulated values, with the median reported as the point estimate. This approach captures parameter uncertainty, and stochastic variation in mortality.

All-cause excess deaths were derived by subtracting the expected death counts from the observed death counts, taken directly from the STMF data. Excess deaths were left-censored at zero.^51^^,^^52^ This was done to ensure a consistent interpretation of sex differences in excess mortality over time. When negative excess values are allowed, the same numerical sex difference can arise from qualitatively different situations—for example, higher excess mortality in one sex, a mortality deficit in the other, or mortality deficits in both sexes of different magnitudes. Left censoring avoids this ambiguity by defining excess mortality strictly as a mortality burden during the pandemic. Under this definition, zero indicates the absence of excess mortality, and positive values indicate additional deaths relative to expected levels. Thus, we defined excess deaths by sex, country, age, and period as the following:

$$Excess\;deaths=\left\{ \begin{array}{c} Observed-Expected,Observed\geq Expected\\ 0,Observed<Expected\text{,} \end{array}\right.$$

and the excess death rate (per 100,000 person-years) as

$$Excess\;death\;rate=\frac{Excess\;Deaths}{Person-years}\times 100,000$$

When reporting results for all age groups combined, we applied direct age standardisation using the 2013 European Standard Population (Eurostat). Across age groups *x* we defined

$$Age\;standardized\;excess\;deaths=\sum_{x}{Excess\;death\;rate}_{x}\times {Standard\;population}_{x}\;\text{.}$$

As a relative measure of excess death, we calculated the so-called P-score, the percentage difference between reported and expected deaths, as:

$$P-\text{score}=\frac{\text{Excess}\;\text{Deaths}}{\text{Expected}\;\text{Deaths}}\times 100$$

Excess mortality estimates were used to calculate both absolute and relative measures of the sex gap. The absolute gap is the difference between male and female all-cause excess death rates. The relative gap is calculated as the percentage point difference between male and female excess mortality P-scores. Further, we calculated expected (under pre-pandemic trends) and observed sex ratios in all-cause mortality.

Due to the large number of age group–country combinations, we discuss age-specific results for six countries reflecting a range of pre-pandemic sex differences in mortality, sociocultural contexts, and pandemic experiences: Bulgaria, England & Wales, Germany, Italy, Norway, and the United States. Results for the full set of countries are available in the Supplementary Material. Among high-income nations, the United States and England & Wales recorded among the highest per capita COVID-19 death rates. Italy faced high COVID-19 mortality early in the pandemic, implemented high levels of pandemic restrictions and has one of the world’s oldest populations. Norway, a high-income economy known for its high life expectancy, despite low levels of restrictions, experienced low overall COVID-19 mortality and a later onset of excess mortality. Germany, another wealthy European nation, implemented strict restrictions and experienced low excess mortality in 2020, followed by high excess in 2021–2023. Bulgaria, an Eastern European nation with large pre-pandemic sex disparities in mortality and one of the highest proportions of older adults in the world,^53^ experienced one of the highest COVID-19 mortality rates in the world.

This analysis relies solely on anonymised, aggregated data that contain no identifying information. All analyses and results are fully reproducible and can be accessed here. We used RStudio (version 2023.9.1.494) for all statistical analyses.

### Patient and public involvement

Patients and the public were not involved in any way in this study.

### Ethics statements

This study used publicly available, de-identified, and aggregated population-level data from the Short-Term Mortality Fluctuations database and the Human Mortality Database. Therefore, ethical approval and informed consent were not required.

### Role of the funding source

The researchers involved in this study declare full independence from the funders. The funding bodies had no role in the design of the study, analysis, or interpretation of the data; the writing of the manuscript; or the decision to submit it for publication. Authors were not precluded from accessing data in the study, and they accept responsibility to submit for publication.

## Results

**Fig. 1:**
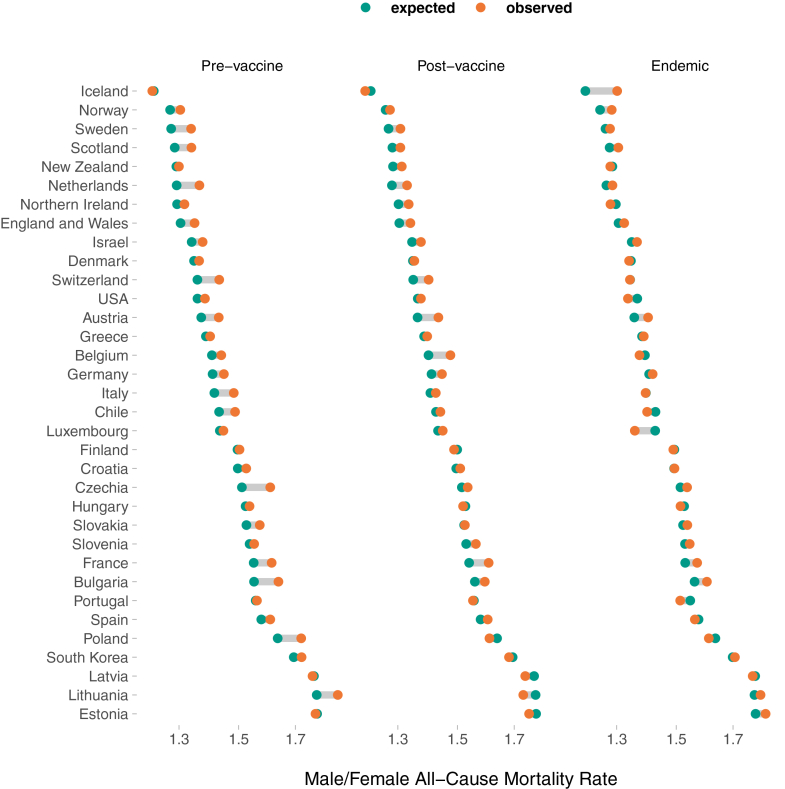
**Sex ratios (male/female) in all-cause mortality by country and pandemic phase, comparing expected and observed mortality.** A ratio of 1 indicates no sex difference. Ratio > 1 indicates higher male mortality. Ratio < 1 indicates higher female mortality. Expected mortality is based on pre-pandemic trends (2013–2019). Pandemic phases: pre-vaccine (February 2020–April 2021), post-vaccine Delta/Omicron (May 2021–April 2022), endemic (May 2022–July 2023).

Fig. 1 shows male-to-female ratios ($\frac{male}{female}$) in expected (green dots) and observed (orange dots) all-cause mortality by period and country, ordered from lowest to highest expected ratios based on pre-pandemic trends. A ratio of 1 means no sex difference in the mortality rate. Ratios above 1 indicate higher male mortality; ratios below 1 indicate higher female mortality. This figure shows the range of cross-country mortality sex differences prior to the pandemic, ranging from small differences in Iceland, Norway, Sweden to large differences in the Baltic countries (Estonia, Lithuania, Latvia). In the pre-vaccine period, the male-to-female ratio of observed mortality was typically larger than the ratio for expected mortality, indicating the male disadvantage was worse than expected based on pre-pandemic mortality. This can be particularly observed for the Czechia, Poland, and Bulgaria. This gap tended to decrease as the pandemic progressed, but the male disadvantage compared to expected mortality ratios persisted for several countries through 2023. In 31 out of 34 countries, sex ratios in observed mortality got smaller during the endemic phase, compared to the pre-vaccine period, with Estonia, Latvia, and Iceland being the exceptions.

**Fig. 2:**
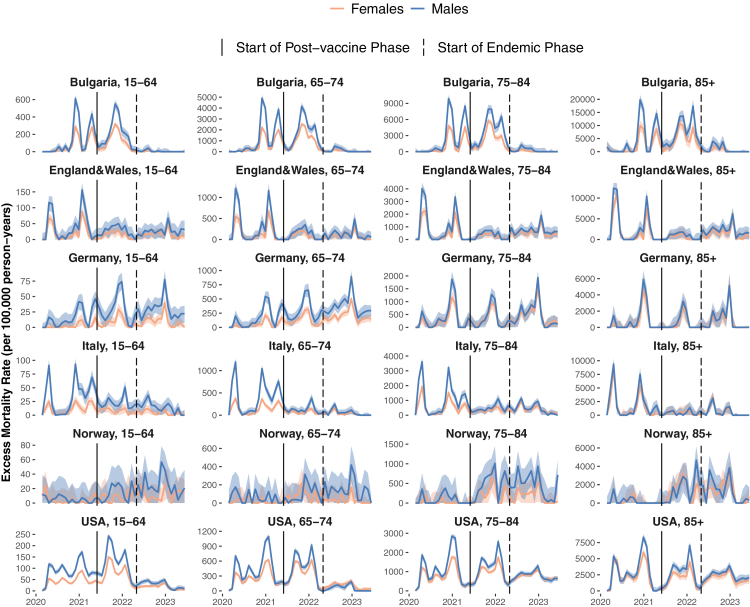
**Monthly trends in all-cause excess mortality death rates (per 100,000 person-years) by sex, age group, and country,February2020–July 2023.** Shaded areas show 95% prediction intervals. The scale of the y-axis differs for each age group and country. Pandemic phases: pre-vaccine (February 2020–April 2021), post-vaccine Delta/Omicron (May 2021–April 2022), endemic (May 2022–July 2023).

Fig. 2 shows monthly trends in all-cause excess mortality death rate per 100,000 person-years by sex between February 2020 and July 2023 across six countries and four age groups (see Supplementary Figs. S1–S5 for the remaining countries). In general, the difference between male and female excess mortality death rate closely followed the overall mortality levels. In months with higher mortality, the sex difference grows, with males seeing higher absolute excess than females. During months with lower mortality, the 95% prediction intervals for male and female excess mortality largely overlap (i.e., no evidence for male disadvantage in these periods).

Within specific countries, Bulgaria and the USA experienced excess mortality peaks with a sizeable male disadvantage in both pre- and post-vaccine phases, but sex differences narrowed with lower mortality levels in the endemic phase. England & Wales and Italy saw large peaks in excess mortality in the pre-vaccine period, with a clear male disadvantage in months where mortality peaked. Germany saw mortality peaks in all three periods, but significant sex gaps were mainly seen under age 75. Norway saw lower mortality early in the pandemic, with peaks occurring later in the endemic phase. In most months, including peak periods, sex differences remained negligible. In Denmark, Finland, Iceland, New Zealand and Northern Ireland, the 95% prediction intervals for male and female excess mortality overlap across all three periods (Supplementary Figs. S1–S3).

**Fig. 3:**
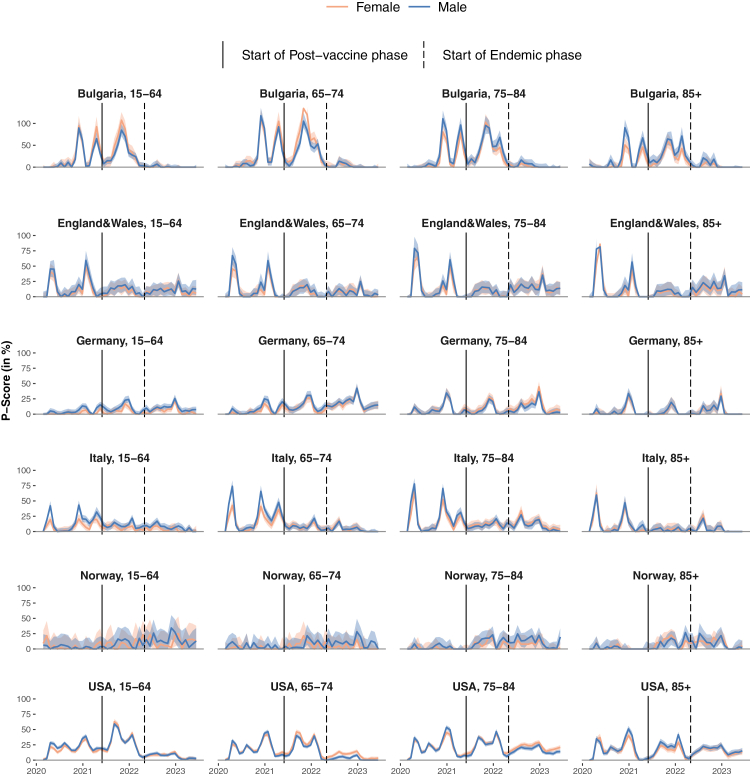
**Monthly trends in all-cause excess mortality P-scores (%) by sex, age group, and country,February2020–July 2023.** P-scores represent percentage deviations from expected all-cause mortality. Shaded areas show 95% prediction intervals. The y-axis scale is fixed across panels (from 0% to +100%), except for Bulgaria (from 0% to +120%), due to higher excess mortality than the remaining five countries. Pandemic phases: pre-vaccine (February 2020–April 2021), post-vaccine Delta/Omicron (May 2021–April 2022), endemic (May 2022–July 2023).

Fig. 3 illustrates monthly trends in P-scores by sex and age between February 2020 and July 2023 for the selected six countries (see Supplementary Figs. S6–S10 for the remaining countries). In contrast to the trends in absolute sex differences in excess mortality (Fig. 2), male disadvantage in P-scores was not clearly observed, regardless of mortality levels (Fig. 3), and in some cases, females had higher relative mortality increases than males (e.g., 65–74 and 75–84-year-olds in the US during the endemic period). Higher female P-scores mean that deaths for women increased by a higher percentage from the baseline (expected mortality) levels compared to those for men.

Overall, males suffered higher absolute levels of excess mortality during pandemic peaks in most countries, especially in the pre-vaccine phase. However, trends in relative mortality suggest that these absolute sex differences largely reflect higher baseline mortality among males, while percentage increases in mortality due to COVID-19 were similar across sexes for most age group–country combinations (Fig. 3).

Given changing biological and social dynamics during the pandemic (e.g., evolution of the COVID-19 virus, introduction of vaccines, changing levels of government lockdowns and restrictions), we next compared excess mortality death rates (Fig. 4), and P-scores (Fig. 5) calculated separately for each sex, age group and period across six countries (Supplementary Figs. S11–S22 for all countries, together with 95% prediction intervals for the difference between male and female estimates).

**Fig. 4:**
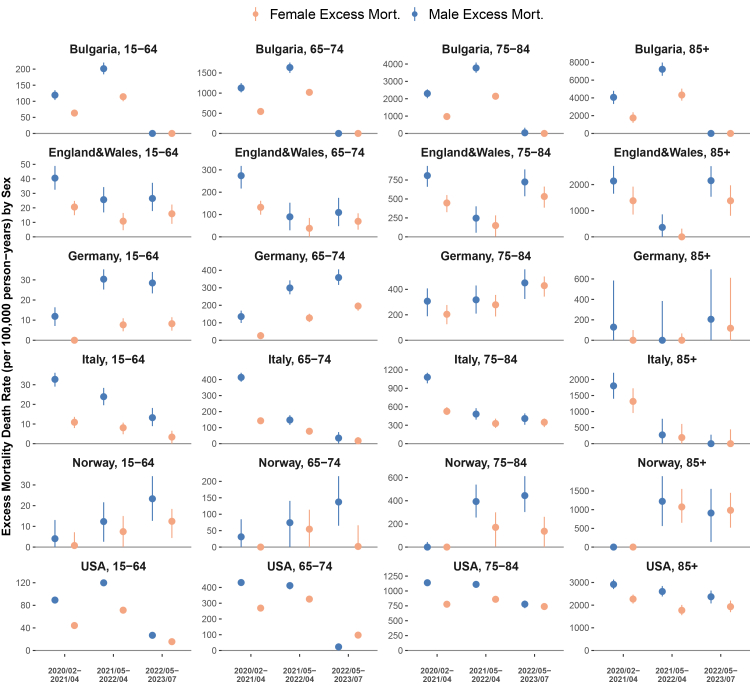
**All-cause excess mortality death rates (per 100,000 person-years) by sex, age group, country, and pandemic phase (pre-vaccine, post-vaccine, endemic).** Bars indicate 95% prediction intervals. Y-axis scales differ by age group and country. Pandemic phases: pre-vaccine (February 2020–April 2021), post-vaccine Delta/Omicron (May 2021–April 2022), endemic (May 2022–July 2023).

**Fig. 5:**
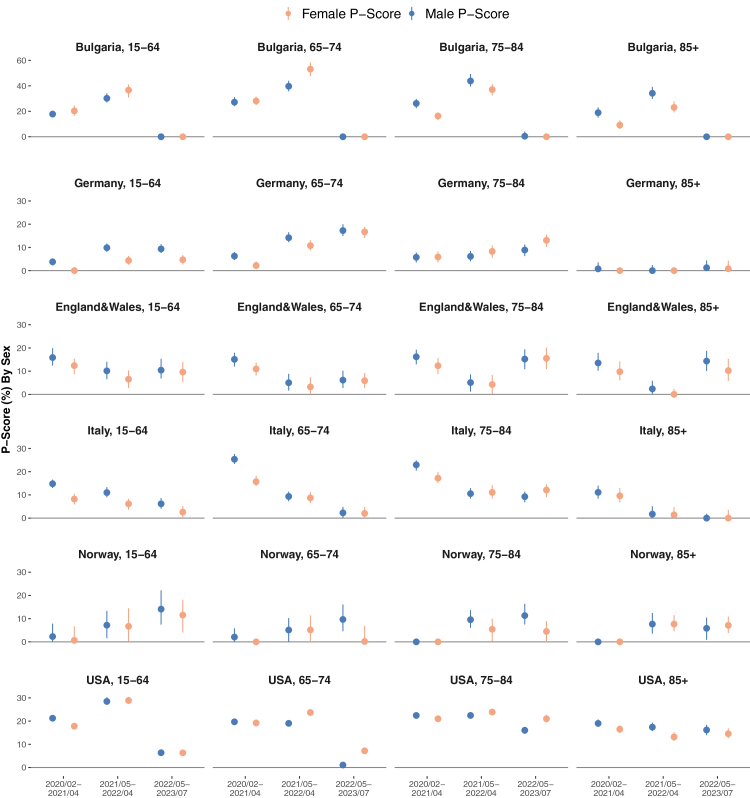
**All-cause excess mortality P-scores (%) by sex, age group, country, and pandemic phase (pre-vaccine, post-vaccine, endemic).** Bars indicate 95% prediction intervals. The y-axis scale is fixed for all countries and age groups except for Bulgaria. Pandemic phases: pre-vaccine (February 2020–April 2021), post-vaccine Delta/Omicron (May 2021–April 2022), endemic (May 2022–July 2023).

A significant male disadvantage was seen in more than half of the age group–country combinations (60%, 81 out of 136) during the pre-vaccine period for excess mortality death rates (Fig. 4 and Supplementary Figs. S11–S16). In the post-vaccine period, this absolute male disadvantage became less common, staying significant in about half of the age group–country combinations (51%, 70 out of 136). By the endemic period, the male disadvantage remained significant in about only one-fifth of age group–country combinations (23%, 31 out of 136); where the remaining combinations showed either no significant sex difference or a significant female disadvantage. There were two exceptions to this pattern: (1) countries with a delayed onset of the pandemic, such as Germany and South Korea, where the male disadvantage increased during the post-vaccine and endemic phases for some age groups (e.g., 15–64 and 65–74-years-olds in Germany), and (2) countries with consistently low levels of male and female excess mortality death rates, such as Iceland, Finland, Luxemburg and New Zealand, where no evidence of male disadvantage was found across most age groups and periods.

Significant male disadvantage during the pre-vaccine phase was much less common when looking at relative sex differences (Fig. 5 and Supplementary Figs. S17–S22), compared to absolute sex differences (Fig. 4). For sex differences in the P-score, in the pre-vaccine phase, a significant male disadvantage was seen in about one-quarter (28%, 38 out of 136) of age group–country combinations; where the remaining combinations showed either no significant sex difference or a significant female disadvantage. In the post-vaccine phase, the relative male disadvantage further decreased, remaining significant in only one in seven (14%, 19 out of 136) of age group–country combinations. In some cases, the P-score was higher for females. For example, a female relative disadvantage emerged in the post-vaccine phase among 65–74-year-olds in Bulgaria and the USA, as well as in other groups. Overall, there was little evidence of remaining relative sex gaps during the endemic phase, with a few exceptions. These exceptions included (1) countries with a delayed pandemic onset, where male relative disadvantage either continued or re-emerged in the endemic phase (e.g., 15–64 group in Germany, 65–74 and 75–84-year-olds in Norway); and (2) some cases of emerging female relative disadvantage (i.e., higher female P-score) in specific age group–country combinations (e.g., 65–74-year-olds in the US and Spain).

Taken together, although men experienced higher absolute all-cause excess mortality during the pre-vaccine phase, both sexes experienced proportionally similar increases in all-cause excess mortality when compared to pre-pandemic levels in most age groups and countries.

Supplementary Figs. S23 and S24 summarise the age-specific patterns in absolute and relative sex differences in all-cause excess mortality by period and country. For absolute sex differences (male minus female excess all-cause mortality death rate per 100,000 person-years; Supplementary Fig. S23), the male disadvantage increases monotonically with age (from 15–64 through to 85+) in just over half of the countries (56%, 19 out of 34) during the pre-vaccine phase. However, this pattern of increasing male disadvantage with age weakens in the later stages of the pandemic.

The age pattern is less clear for relative sex differences (male minus female P-score; Supplementary Fig. S24). While males generally had higher P-scores across most age group–country combinations during the pre-vaccine period, several age group–country combinations shifted towards a female disadvantage in later periods.

## Discussion

This study provides a comprehensive analysis of sex differences in pandemic-related all-cause excess mortality during the COVID-19 pandemic in 34 high-income countries, covering the pandemic’s progression through its pre-vaccine, post-vaccine, and endemic phases. Unlike earlier studies, we use an excess mortality framework, avoiding potential biases introduced by variability in testing and recording of COVID-19 deaths across countries. All-cause excess mortality captures both direct COVID-19 deaths and indirect deaths resulting from healthcare disruption, behavioural change, and socio-economic stressors. Our findings show that the COVID-19 pandemic temporarily amplified pre-existing sex differences in all-cause mortality. Specifically, the observed male-to-female mortality ratios were higher than expected based on pre-pandemic trends, indicating a greater-than-usual male disadvantage during the pre-vaccine phase of the pandemic. In the endemic phase, observed ratios declined, becoming only slightly higher than expected—or, in some cases, even falling below them. This short-lived pattern of elevated sex differences in excess mortality contrasts with the 1918 influenza pandemic, which, had a longer lasting impact on sex differences in mortality. The 1918 flu caused unusually high male mortality, possibly due to co-infection with tuberculosis, which was more prevalent among men. Noymer and Garenne hypothesise that the reduction in the sex differences in mortality in the US in the years following the pandemic was due to strong mortality selection that left behind cohorts of relatively healthier males.^33^ Our results suggest that COVID-19 is unlikely to produce similar long-term effects on sex differences in mortality in high-income countries.

Earlier studies consistently reported a male disadvantage in COVID-19 mortality.^19^, ^20^, ^21^^,^^23^, ^24^, ^25^, ^26^, ^27^, ^28^, ^29^, ^30^^,^^32^ Our analysis of both absolute and relative sex gaps over time reveals a more nuanced picture. In absolute terms, and in line with earlier research, males experienced higher excess mortality during peak months, often exceeding pre-pandemic male-female mortality differences. However, this was not a universal pattern; even in the pre-vaccine period, many age group–country combinations showed no significant male disadvantage. In contrast, relative measures showed less consistent male disadvantage. In several age group–country combinations, females had a greater percentage increase in excess mortality relative to expected deaths, especially after the vaccine rollout. By the endemic phase, relative sex gaps had diminished across most countries and age groups. These reductions were often driven by steeper declines in male excess mortality, though in some cases, increased female mortality also contributed to the narrowing gap.

A key reason why sex differences appear smaller when using relative rather than absolute mortality is that relative measures naturally account for men’s higher baseline mortality at almost every age. When absolute deaths rise during the pandemic, the male disadvantage looks larger partly because it reflects the same longstanding structural gap seen in normal all-cause mortality. Relative mortality, by contrast, scales COVID-19 deaths to each sex’s underlying risk. This helps isolate the additional hazard that COVID-19 introduces, rather than conflating the effect of the virus with the pre-existing male disadvantage. This pattern is consistent with work showing that COVID-19 mortality tends to rise in step with “normal” age-related risks.^54^ If COVID-19 amplifies the existing mortality structure rather than creating a new one, then standardising by baseline risk will make the sex gap appear more stable.

Several mechanisms may further explain the observed sex differences in direct COVID-19 mortality. We organise these into three categories: (i) risk of exposure to the virus, (ii) risk of becoming infected given exposure, and (iii) risk of dying given infection.

Sex differences in exposure are shaped primarily by social and behavioural factors, including occupation, mobility, and adherence to public health measures. Previous studies have emphasised the strong connection between occupational roles and exposure to the COVID-19 virus.^55^ In many countries, female-dominated sectors such as education and administrative work were more likely to shift to remote work.^56^ Conversely, remote work was less feasible in male-dominated industries such as agriculture, security, logistics, construction, food production and manufacturing^56^ -- sectors that reported some of the largest increases in mortality during the pandemic.^57^ This pattern may explain the male disadvantage observed during the pre-vaccine phase. At the same time, women are overrepresented in frontline healthcare, care, and retail occupations, which carried high exposure risk.^58^, ^59^, ^60^ This likely contributed to the absence of a universal male disadvantage across countries and age groups.

Behavioural sex differences may also have influenced exposure. Survey evidence shows that men were, on average, less likely to adhere to public health recommendations such as mask-wearing, hand washing, and social distancing,^34^^,^^35^ and reported lower perceived risk of contracting COVID-19.^61^^,^^62^ These behaviours may have increased exposure among men.

The risk of becoming infected given exposure reflects both biological susceptibility^9^, ^10^, ^11^, ^12^, ^13^, ^14^^,^^16^^,^^17^ and vaccine-induced immunity. Biologically, women tend to exhibit stronger and longer-lasting immune responses than men, and these responses decline less with age in women. This may help explain greater male vulnerability early in the pandemic, when populations were largely immune-naive. At the same time, COVID-19 vaccine uptake differed by sex. Surveys conducted in Europe and the US showed higher vaccine hesitancy and lower uptake among women.^63^^,^^64^ This could help to explain the faster decline in male mortality and the narrowing of sex gaps in later phases of the pandemic.

Additional factors may also have contributed to the faster decline in male mortality. Mortality displacement could have played a role if more frail men died earlier in the pandemic, leaving a healthier surviving population. Improvements in workplace protections and other public health measures in male-dominated industries may also have reduced exposure and mortality risk over time, thereby narrowing or closing the sex gap in the post-vaccine and endemic phases.

Finally, sex differences in the risk of dying given infection are shaped by immune function and underlying health. Women’s stronger innate and adaptive immune responses—driven by oestrogen and genetic factors linked to the double X chromosome—are thought to reduce the risk of severe disease and death once infected.^15^^,^^16^ These immune differences may help explain the male disadvantage observed during periods of high mortality, particularly early in the pandemic.

Sex differences in occupational exposure and protective behaviours likely interacted with policy environments to shape cross-country variation in mortality differentials. Countries differed in the timing, duration and strictness of workplace safety rules, including the availability and enforcement of personal protective equipment, ventilation standards, physical-distancing requirements, sick-leave provisions, and vaccine mandates.^60^^,^^65^ These policy differences likely moderated how much occupational gender segregation translated into unequal exposure risk. More broadly, longer durations of distancing policies were associated with more frequent protective behaviours among citizens.^66^ Such policies reduced reliance on individual risk assessment and personal choice. As a result, sex differences in adherence to protective behaviours, both in and outside of the workplace, may have mattered less for infection risk in more regulated contexts. By contrast, in settings with weaker enforcement or lower baseline compliance, behavioural differences were more likely to translate into differential exposure and mortality risks.

Higher vaccine hesitancy among women, observed in several countries,^67^^,^^68^ may have been amplified in contexts characterised by mistrust in government.^69^ Countries also differed in vaccination policies, including the stringency of mandates, the use of financial incentives, and effectiveness of communication strategies.^67^^,^^70^ These differences likely shaped both sex gaps in hesitancy and uptake. Taken together, these patterns suggest that sex differences in social and behavioural risk factors were context-dependent and mattered less in policy environments that standardised exposure risks, providing a plausible explanation for the substantial cross-country heterogeneity in sex differences in excess mortality observed in our study.

Some of the weaker or reversed relative sex differences observed in later phases may reflect gendered indirect consequences of the pandemic rather than shifts in COVID-19 mortality itself. First, disruptions to women’s health services and declines in preventive care likely increased non-COVID health risks for women in some countries.^71^, ^72^, ^73^, ^74^ Second, women were more exposed to employment and income shocks because they were overrepresented in the sectors hit hardest by lockdowns.^75^^,^^76^ Third, school and childcare closures increased unpaid care demands that fell disproportionately on women,^77^ perhaps especially in countries with weaker public care infrastructures. Finally, women faced higher levels of psychological distress and greater exposure to domestic violence during lockdowns.^78^^,^^79^ These indirect pathways, which vary substantially across national policy environments, offer plausible explanations for occasional higher female P-scores.

We observed substantial regional variation in the magnitude and age patterns of both absolute and relative sex differences in excess mortality, reflecting differences in social, economic, and healthcare contexts. In the pre-vaccine phase, both absolute and relative sex differences were among the largest in Eastern Europe (see Supplementary Tables S1 and S2), consistent with pre-pandemic sex differences in all-cause mortality of some Eastern European countries,^80^ which the pandemic amplified. High levels of smoking and chronic drinking among Eastern European men, factors associated with greater risk of COVID-19 infection and severe disease progression^81^^,^^82^—may have contributed to elevated excess mortality in this group and also underlie much of the pre-pandemic sex gap in cardiovascular disease and lung cancer mortality.^83^^,^^84^ Cross-country differences may further reflect regional variation in occupational gender segregation,^85^^,^^86^ which has declined substantially in Scandinavia in recent decades, but remained high or increased in parts of Eastern and Mediterranean Europe,^85^ potentially contributing to larger sex differences in pandemic mortality in these regions.

The mechanisms outlined are intended to frame plausible sources of variation rather than to assert causality. As our excess mortality approach cannot distinguish direct COVID-19 deaths from indirect consequences, these explanations should be read as informed hypotheses rather than empirically verified drivers.

Our findings have implications for policy and practice. The large cross-country differences in the size and direction of sex gaps in all-cause excess mortality during the COVID-19 pandemic underscore the importance of adopting a gender lens in health-system planning and preparedness. These patterns highlight the role of social and policy contexts in shaping sex-specific vulnerability and indicate that pandemics tend to exacerbate pre-existing inequalities, including gender inequalities. Our findings also highlight the importance of employing both absolute and relative metrics in crisis surveillance. Absolute measures capture the overall mortality burden and pressure on health systems, whereas relative measures contextualise crisis impacts against baseline inequalities and can reveal disproportionate effects that are not visible in death counts alone. Finally, effective preparedness and response strategies should monitor both direct and indirect effects of pandemics using timely, sex- and age-disaggregated epidemiological data. The continued absence of such data for many countries points to a critical global surveillance gap that needs to be addressed to enable more equitable and evidence-based responses to future health crises.

Our analysis has limitations. First, our conclusions are limited to the selected high-income countries with efficient vital registration systems. As a result, our findings should not be generalised to low- and middle-income countries, where patterns of sex differences in excess mortality may differ due to distinct population age structures, health profiles, healthcare systems, and social and economic inequalities. Second, our population counts for 2022–2023 were extrapolated from 2021. This may lead to bias due to not accounting for pandemic deaths and migration. Third, the STMF data is available only for very broad age groups, most notably the 15–64 category, which combines adults at very different stages of the life course. For this reason, we were able to only roughly account for between-country differences in age structure.^87^ The 15–64 grouping also limits our ability to examine heterogeneity within the working-age populations, such as differences between younger and older adults. Although the finer age stratification would be desirable, further disaggregation of weekly mortality data would require strong assumptions that may not hold during acute mortality shocks, particularly given the pronounced concentration of COVID-19 mortality at older ages.^49^ Our age-specific findings should therefore be interpreted as conservative summaries within the constraints of harmonised cross-national data. Fourth, while the all-cause excess mortality framework ensures comparability across countries and over time, it combines deaths directly attributable to COVID-19 with indirect deaths resulting from the broader consequences of the pandemic (e.g., mental health crises or healthcare disruptions). Absolute and relative sex differences in these indirect deaths may exhibit different directions, age and temporal patterns than those reported here. However, available evidence suggests that misclassification of COVID-19 deaths likely accounts for a substantial share of non-COVID excess mortality, indicating that indirect causes represent a relatively smaller component of the overall excess death burden.^48^ Fifth, by left-censoring excess deaths at zero, the analysis does not capture periods of mortality deficit or potential mortality displacement (‘harvesting’), particularly in the endemic phase. Our focus, however, is on comparing sex differences in excess mortality attributable to the pandemic shock itself, rather than on compensation dynamics. Finally, our data does not account for gender identity and is available only for people categorised as women and men.

We present a comprehensive analysis of sex differences in excess mortality during the COVID-19 pandemic, covering 34 high-income countries across three phases: pre-vaccine, post-vaccine, and endemic. Addressing limitations of earlier studies, we use an excess mortality framework and examine both absolute and relative measures of sex differences, allowing for more reliable comparisons across time and place. While the prevailing narrative highlights a persistent male disadvantage in COVID-19 mortality, our findings show that this pattern was context-specific. Male disadvantage was evident during high-mortality months in the pre-vaccine phase and in absolute terms, but it was not universal. When using relative measures, which account for baseline mortality differences, the male disadvantage largely disappeared—or even reversed—in most age group–country combinations. These findings underscore the importance of using both absolute and relative metrics to understand inequalities in mortality and suggest that COVID-19 did not produce lasting shifts in sex differences in mortality.

## Contributors

KD and JBD conceived and designed the study. JS curated and prepared the data. KD carried out the statistical analysis and created the figures. JBD and JS reviewed the analysis. KD wrote the first draft of the manuscript. KD, JBD, JS, and MK contributed to the interpretation of the findings and to the critical revision of the manuscript. KD and JS directly accessed and verified the underlying data and verified the analyses. All authors read and approved the final version of the manuscript. KD is the guarantor and accepts full responsibility for the overall content, had full access to the data, and controlled the decision to publish. The corresponding author attests that all listed authors meet authorship criteria and that no others meeting the criteria have been omitted.

## Data sharing statement

Original data used in the analyses of this manuscript is publicly available at https://www.mortality.org/Data/STMF. Processed data and all analyses and results are fully reproducible and can be accessed here :https://osf.io/wmt85.

## Declaration of interests

All authors have completed the ICMJE disclosure form. We declare no competing interests.
