# Supplementary material for: Sex differences in excess mortality during the COVID-19 pandemic: a longitudinal ecological analysis of 34 high-income countries
Source: eClinicalMedicine. 2026 Jul 23;98:104072. doi: 10.1016/j.eclinm.2026.104072 (PMC13427654; doi:10.1016/j.eclinm.2026.104072)
Supplement: Supplementary Figs. S1–S24 and Tables S1 and S2 [file mmc1.docx]

Supplementary

Material

**Supplement to:** Sex differences in excess mortality during the COVID-19 pandemic: a longitudinal ecological analysis of 34 high-income countries

**Table of Contents**

[*Supplementary Figures 4*](#_Toc223471737)

[*Supplementary Tables 27*](#_Toc223471738)

**Table of Figures and Tables**

[Figure S 1. Monthly trends in excess mortality death rate (per 100,000 person-years) by sex, age, and country [Austria, Belgium, Croatia, Czechia, Denmark, and Hungary]. Part 1 of 5.. 4](#_Toc223952900)

[Figure S 2. Monthly trends in excess mortality death rate (per 100,000 person-years) by sex, age, and country [Estonia, Finland, France, Greece, Iceland, and Israel]. Part 2 of 5. 5](#_Toc223952901)

[Figure S 3. Monthly trends in excess mortality death rate (per 100,000 person-years) by sex, age, and country [Latvia, Lithuania, Luxembourg, Netherlands, New Zealand, and Northern Ireland]. Part 3 of 5. 6](#_Toc223952902)

[Figure S 4. Monthly trends in excess mortality death rate (per 100,000 person-years) by sex, age, and country [Poland, Portugal, Scotland, Slovakia, Slovenia and South Korea]. Part 4 of 5. 7](#_Toc223952903)

[Figure S 5. Monthly trends in excess mortality death rate (per 100,000 person-years) by sex, age, and country [Chile, Spain, Sweden, and Switzerland]. Part 5 of 5. 8](#_Toc223952904)

[Figure S 6. Monthly trends in excess death P-scores by sex, age and country [Austria, Belgium, Croatia, Czechia, Denmark, and Hungary]. Part 1 of 5. 9](#_Toc223952905)

[Figure S 7. Monthly trends in excess death P-scores by sex, age and country [Estonia, Finland, France, Greece, Iceland, and Israel]. Part 2 of 5. 10](#_Toc223952906)

[Figure S 8. Monthly trends in excess death P-scores by sex, age and country [Latvia, Lithuania, Luxembourg, Netherlands, New Zealand, and Northern Ireland]. Part 3 of 5. 11](#_Toc223952907)

[Figure S 9. Monthly trends in excess death P-scores by sex, age and country [Poland, Portugal, Scotland, Slovakia, Slovenia and South Korea]. Part 4 of 5. 12](#_Toc223952908)

[Figure S 10. Monthly trends in excess death P-scores by sex, age and country [Chile, Spain, Sweden, and Switzerland]. Part 5 of 5. 13](#_Toc223952909)

[Figure S 11. All-cause excess mortality rate by sex (left y-axis) and the sex gap in excess mortality (male minus female, right y-axis), shown by age group, period, and country [Bulgaria, England&Wales, Germany, Italy, Norway, and USA]. Part 1 of 6. 14](#_Toc223952910)

[Figure S 12. All-cause excess mortality rate by sex (left y-axis) and the sex gap in excess mortality (male minus female, right y-axis), shown by age group, period, and country [Austria, Belgium, Croatia, Czechia, Denmark, and Hungary]. Part 2 of 6. 15](#_Toc223952911)

[Figure S 13. All-cause excess mortality rate by sex (left y-axis) and the sex gap in excess mortality (male minus female, right y-axis), shown by age group, period, and country [Estonia, Finland, France, Greece, Iceland, and Israel]. Part 3 of 6. 16](#_Toc223952912)

[Figure S 14. All-cause excess mortality rate by sex (left y-axis) and the sex gap in excess mortality (male minus female, right y-axis), shown by age group, period, and country [Latvia, Lithuania, Luxembourg, Netherlands, New Zealand, and Northern Ireland]. Part 4 of 6. 17](#_Toc223952913)

[Figure S 15. All-cause excess mortality rate by sex (left y-axis) and the sex gap in excess mortality (male minus female, right y-axis), shown by age group, period, and country [Poland, Portugal, Scotland, Slovakia, Slovenia and South Korea]. Part 5 of 6. 18](#_Toc223952914)

[Figure S 16. All-cause excess mortality rate by sex (left y-axis) and the sex gap in excess mortality (male minus female, right y-axis), shown by age group, period, and country [Chile, Spain, Sweden, and Switzerland]. Part 6 of 6. 19](#_Toc223952915)

[Figure S 17. Excess death P-score by sex (left y-axis) and the sex gap in P-score (male minus female, right y-axis), shown by age group, period, and country [Bulgaria, England&Wales, Germany, Italy, Norway, USA]. Part 1 of 6. 20](#_Toc223952916)

[Figure S 18. Excess death P-score by sex (left y-axis) and the sex gap in P-score (male minus female, right y-axis), shown by age group, period, and country [Austria, Belgium, Croatia, Czechia, Denmark, and Hungary]. Part 2 of 6. 21](#_Toc223952917)

[Figure S 19. Excess death P-score by sex (left y-axis) and the sex gap in P-score (male minus female, right y-axis), shown by age group, period, and country [Estonia, Finland, France, Greece, Iceland, Israel]. Part 3 of 6. 22](#_Toc223952918)

[Figure S 20. Excess death P-score by sex (left y-axis) and the sex gap in P-score (male minus female, right y-axis), shown by age group, period, and country [Latvia, Lithuania, Luxembourg, Netherlands, New Zealand, Northern Ireland]. Part 4 of 6. 23](#_Toc223952919)

[Figure S 21. Excess death P-score by sex (left y-axis) and the sex gap in P-score (male minus female, right y-axis), shown by age group, period, and country [Poland, Portugal, Scotland, Slovakia, Slovenia and South Korea]. Part 5 of 6. 24](#_Toc223952920)

[Figure S 22. Excess death P-score by sex (left y-axis) and the sex gap in P-score (male minus female, right y-axis), shown by age group, period, and country [Chile, Spain, Sweden, and Switzerland]. Part 6 of 6. 25](#_Toc223952921)

[Figure S 23. Sex gap (male-female) in excess all-cause mortality death rate (per 100,000 person-years) by age group, period and country. 26](#_Toc223952922)

[Figure S 24. Sex gap (male-female) in P-score by age group, period and country. 26](#_Toc223952923)

[Table S 1. Absolute sex gap in excess mortality (per 100,000 person‑years). Male minus female excess death rate per 100,000 person-years. Values are median (2.5% PI, 97.5% PI), except for single‑country entries. 26](#_Toc223471208)

[Table S 2. Relative sex gap in excess mortality (%pt). Percentage point difference in male minus female excess mortality P-scores. Values are median (2.5% PI, 97.5% PI). 26](#_Toc223471209)

# *Supplementary Figures*

Figure S 1. Monthly trends in excess mortality death rate (per 100,000 person-years) by sex, age, and country [Austria, Belgium, Croatia, Czechia, Denmark, and Hungary]. Part 1 of 5..

Figure S 2. Monthly trends in excess mortality death rate (per 100,000 person-years) by sex, age, and country [Estonia, Finland, France, Greece, Iceland, and Israel]. Part 2 of 5.

Figure S 3. Monthly trends in excess mortality death rate (per 100,000 person-years) by sex, age, and country [Latvia, Lithuania, Luxembourg, Netherlands, New Zealand, and Northern Ireland]. Part 3 of 5.

Figure S 4. Monthly trends in excess mortality death rate (per 100,000 person-years) by sex, age, and country [Poland, Portugal, Scotland, Slovakia, Slovenia and South Korea]. Part 4 of 5.

Figure S 5. Monthly trends in excess mortality death rate (per 100,000 person-years) by sex, age, and country [Chile, Spain, Sweden, and Switzerland]. Part 5 of 5.

Figure S 6. Monthly trends in excess death P-scores by sex, age and country [Austria, Belgium, Croatia, Czechia, Denmark, and Hungary]. Part 1 of 5.

Figure S 7. Monthly trends in excess death P-scores by sex, age and country [Estonia, Finland, France, Greece, Iceland, and Israel]. Part 2 of 5.

Figure S 8. Monthly trends in excess death P-scores by sex, age and country [Latvia, Lithuania, Luxembourg, Netherlands, New Zealand, and Northern Ireland]. Part 3 of 5.

Figure S 9. Monthly trends in excess death P-scores by sex, age and country [Poland, Portugal, Scotland, Slovakia, Slovenia and South Korea]. Part 4 of 5.

Figure S 10. Monthly trends in excess death P-scores by sex, age and country [Chile, Spain, Sweden, and Switzerland]. Part 5 of 5.

Figure S 11. All-cause excess mortality rate by sex (left y-axis) and the sex gap in excess mortality (male minus female, right y-axis), shown by age group, period, and country [Bulgaria, England&Wales, Germany, Italy, Norway, and USA]. Part 1 of 6.

Figure S 12. All-cause excess mortality rate by sex (left y-axis) and the sex gap in excess mortality (male minus female, right y-axis), shown by age group, period, and country [Austria, Belgium, Croatia, Czechia, Denmark, and Hungary]. Part 2 of 6.

Figure S 13. All-cause excess mortality rate by sex (left y-axis) and the sex gap in excess mortality (male minus female, right y-axis), shown by age group, period, and country [Estonia, Finland, France, Greece, Iceland, and Israel]. Part 3 of 6.

Figure S 14. All-cause excess mortality rate by sex (left y-axis) and the sex gap in excess mortality (male minus female, right y-axis), shown by age group, period, and country [Latvia, Lithuania, Luxembourg, Netherlands, New Zealand, and Northern Ireland]. Part 4 of 6.

Figure S 15. All-cause excess mortality rate by sex (left y-axis) and the sex gap in excess mortality (male minus female, right y-axis), shown by age group, period, and country [Poland, Portugal, Scotland, Slovakia, Slovenia and South Korea]. Part 5 of 6.

Figure S 16. All-cause excess mortality rate by sex (left y-axis) and the sex gap in excess mortality (male minus female, right y-axis), shown by age group, period, and country [Chile, Spain, Sweden, and Switzerland]. Part 6 of 6.

Figure S 17. Excess death P-score by sex (left y-axis) and the sex gap in P-score (male minus female, right y-axis), shown by age group, period, and country [Bulgaria, England&Wales, Germany, Italy, Norway, USA]. Part 1 of 6.

Figure S 18. Excess death P-score by sex (left y-axis) and the sex gap in P-score (male minus female, right y-axis), shown by age group, period, and country [Austria, Belgium, Croatia, Czechia, Denmark, and Hungary]. Part 2 of 6.

Figure S 19. Excess death P-score by sex (left y-axis) and the sex gap in P-score (male minus female, right y-axis), shown by age group, period, and country [Estonia, Finland, France, Greece, Iceland, Israel]. Part 3 of 6.

Figure S 20. Excess death P-score by sex (left y-axis) and the sex gap in P-score (male minus female, right y-axis), shown by age group, period, and country [Latvia, Lithuania, Luxembourg, Netherlands, New Zealand, Northern Ireland]. Part 4 of 6.

Figure S 21. Excess death P-score by sex (left y-axis) and the sex gap in P-score (male minus female, right y-axis), shown by age group, period, and country [Poland, Portugal, Scotland, Slovakia, Slovenia and South Korea]. Part 5 of 6.

Figure S 22. Excess death P-score by sex (left y-axis) and the sex gap in P-score (male minus female, right y-axis), shown by age group, period, and country [Chile, Spain, Sweden, and Switzerland]. Part 6 of 6.

Figure S 23. Sex gap (male-female) in excess all-cause mortality death rate (per 100,000 person-years) by age group, period and country.

Figure S 24. Sex gap (male-female) in P-score by age group, period and country.

# *Supplementary Tables*

Table S 1. Absolute sex gap in excess mortality (per 100,000 person‑years). Male minus female excess death rate per 100,000 person-years. Values are median (2.5% PI, 97.5% PI), except for single‑country entries.

| **Region Group** | **No. Countries** | **Pre-vaccine** | **Post-vaccine** | **Endemic** |
| --- | --- | --- | --- | --- |
| **Northern Europe** | 11 | 35 (20, 50) | 20 (6, 37) | 15 (-2, 29) |
| **Western Europe** | 7 | 36 (23, 48) | 40 (30, 54) | 16 (4, 29) |
| **Southern Europe** | 6 | 38 (24, 51) | 21 (5, 34) | -1 (-15, 16) |
| **Eastern Europe** | 5 | 76 (66, 88) | 13 (0, 30) | -6 (-20, 10) |
| **Chile** | 1 | 48 (33, 59) | 34 (13, 51) | -1 (-22, 23) |
| **Israel** | 1 | 17 (5, 29) | 11 (-1, 26) | 4 (-10, 20) |
| **New Zealand** | 1 | 0 (0, 0) | 2 (-3, 16) | -1 (-26, 27) |
| **South Korea** | 1 | 0 (0, 1) | 3 (-7, 14) | 12 (-1, 26) |
| **United States** | 1 | 37 (31, 44) | 35 (25, 43) | -19 (-29, -9) |

.

Table S 2. Relative sex gap in excess mortality (%pt). Percentage point difference in male minus female excess mortality P-scores. Values are median (2.5% PI, 97.5% PI).

| **Region Group** | **No. Countries** | **Pre-vaccine** | **Post-vaccine** | **Endemic** |
| --- | --- | --- | --- | --- |
| **Northern Europe** | 11 | 3.7 (1.9, 5.4) | 2.1 (0.5, 4.0) | 1.5 (-0.6, 3.2) |
| **Western Europe** | 7 | 3.5 (2.2, 4.7) | 3.9 (2.8, 5.3) | 1.6 (0.3, 2.8) |
| **Southern Europe** | 6 | 3.5 (2.0, 4.8) | 1.9 (0.3, 3.2) | -0.1 (-1.6, 1.5) |
| **Eastern Europe** | 5 | 4.5 (3.4, 5.8) | -0.8 (-2.2, 0.9) | -0.6 (-1.8, 0.8) |
| **Chile** | 1 | 5.2 (2.3, 7.5) | 2.3 (-1.6, 5.7) | -1.7 (-5.3, 2.3) |
| **Israel** | 1 | 3.1 (0.6, 5.6) | 1.9 (-1.0, 5.1) | 0.7 (-2.3, 4.1) |
| **New Zealand** | 1 | 0.0 (0.0, 0.0) | 0.2 (-0.5, 2.3) | -0.4 (-4.0, 3.7) |
| **South Korea** | 1 | 0.0 (0.0, 0.2) | -1.3 (-3.3, 0.7) | 0.6 (-1.6, 3.1) |
| **United States** | 1 | 2.3 (1.4, 3.2) | 1.5 (0.2, 2.7) | -3.8 (-5.1, -2.5) |
